# Supplementary material for: Towards a transferable fermionic neural wavefunction for molecules
Source: Nat Commun. 2024 Jan 2;15:120. doi: 10.1038/s41467-023-44216-9 (PMC10762074; doi:10.1038/s41467-023-44216-9)
Supplement: Supplementary file 1 — Suppplementary Information [file 41467_2023_44216_MOESM1_ESM.pdf]

Supplementary Information - Towards a Transferable Fermionic Neural Wavefunction for Molecules

Supplementary Note 1. Accuracy for single molecules

We compare in Fig. 1 our newly proposed orbital ansatz against other DL-VMC methods when optimizing a single geometry without initialization from a pre-trained base model. We find empirically that our method, using the built-in prior of local orbital coefficients, is able to reach chemical accuracy compared to a high-accurate PsiFormer calculation [1] with a final error of $\sim$ 1mHa. On the other hand PauliNet-like architectures, with ab-initio orbitals as initial prior, cannot reach this reference energy. We compare against two versions of PauliNet [2] implemented in [3]: A re-implementation of the original PauliNet (with minimally modified embedding) and an improved version using an additional backflow shift. When comparing to Gerard et al. [4], which uses the same electron embedding architecture as this work, we obtain a 0.7 mHa higher energy. This might be caused by the newly introduced orbitals, allowing to optimize across molecules but with a slight trade-off for single-molecule accuracy.

Supplementary Note 2. Expressiveness of our ansatz

In the following, we sketch out the theoretical expressiveness of our ansatz, relative to other DL-VMC ansätze. Conventional, non-transferable ansätze such as FermiNet or PsiFormer have the following structure

$$\phi_{ik}^{\text{FN}} = \sum_{I=1}^{N_{\text{nuc}}} \sum_{\nu=1}^{D_{\text{emb}}} w_{k\nu} h_{i\nu} \pi_{Ik} e^{-\omega_{Ik} |\boldsymbol{r}_i - \boldsymbol{R}_I|}, \tag{1}$$

where $w_{k\nu}$, π_{Ik}, $\omega_{Ik} \in \mathbb{R}$ are arbitrary trainable parameters. In comparison, our ansatz has the form

$$\phi_{ik}^{\text{TAO}} = \sum_{I=1}^{N_{\text{nuc}}} \sum_{\nu=1}^{D_{\text{emb}}} f_\nu(\boldsymbol{c}_{Ik}) h_{i\nu} e^{-g(\boldsymbol{c}_{Ik}) |\boldsymbol{r}_i - \boldsymbol{R}_I|}, \tag{2}$$

leading to the following equivalence:

$$f_\nu(\boldsymbol{c}_{Ik}) = w_{k\nu} \pi_{Ik} \tag{3}$$

$$g(\boldsymbol{c}_{Ik}) = \omega_{Ik}. \tag{4}$$

Since neural networks are universal function approximators, this equivalence can be satisfied, for arbitrary choices of $\boldsymbol{w}$, $\boldsymbol{\omega}$, $\boldsymbol{\pi}$, as long as the inputs $\boldsymbol{c}_{Ik}$ are distinct for each index tuple (I, k).

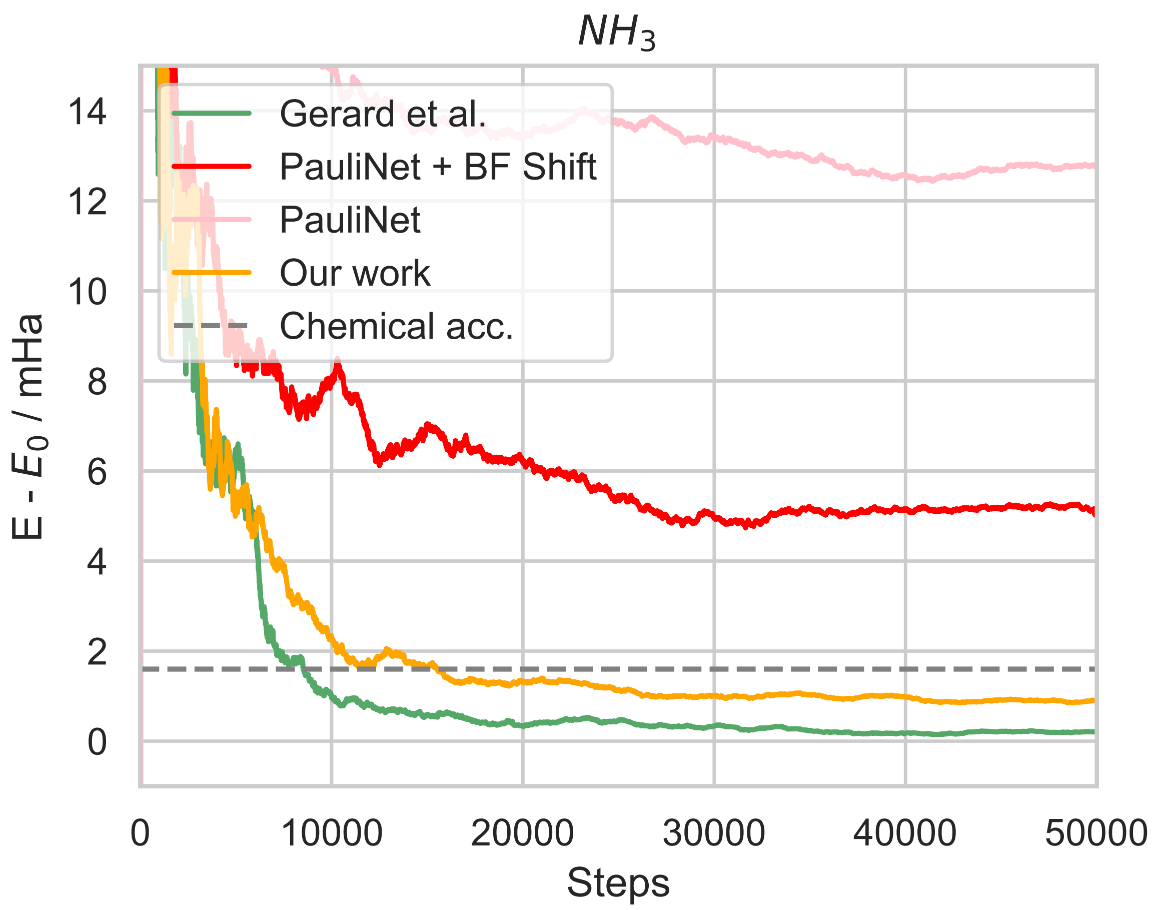

Supplementary Figure 1: Comparison of our work with HF-pre-training (i.e. no initialization from the base model) against other DL-VMC methods [2, 3, 4] for NH_3. PsiFormer [1] is used as a baseline E_0.

The orbital descriptors $\boldsymbol{c}_{Ik}$ are in turn functions of the localized Hartree-Fock coefficients $\widetilde{\boldsymbol{\alpha}}_{Ik}$

$$\boldsymbol{c}_{Ik} = \mathrm{GCN}_I\left(\{\widetilde{\boldsymbol{\alpha}}_{Jk}\}_{J=1\dots N_{\mathrm{nuc}}}, \{\boldsymbol{R}_{JJ'}\}\right), \quad J, J' = 1 \dots N_{\mathrm{nuc}} \tag{5}$$

For single atoms, the Hartree-Fock orbital coefficients $\boldsymbol{\alpha}_k$ are always unique. In fact they are even orthogonal, since they are solutions to a generalized eigenvalue problem. Thus also $\boldsymbol{c}_k$ can be unique, and thus arbitrary backflows and exponents can be expressed. For a general molecule with no geometrical symmetry, unique orbital descriptors $\boldsymbol{c}_{Ik}$ can in principle also be achieved due to the expressiveness of the GCN. Since it can in principle learn any permutation equivariant function, it can output distinct coefficients $\boldsymbol{c}_{Ik}$ as long as all atoms are distinguishable either by geometry or by the orbital coefficients $\boldsymbol{\alpha}$. This only leaves the case of symmetrical molecules, in which indistinguishable atoms exist, for example dimers. In this case non-transferable approaches such as FermiNet can express wavefunctions, which cannot be expressed by our ansatz. In particular,

these are wavefunctions which are not invariant to permutation of the nuclei. Since our ansatz is by design invariant to permutation of atoms (since the Hartree-Fock solution is equivariant to permutation and the sum over I ultimately leads to invariance), it cannot represent these wavefunctions. However, no physical observable can depend on the ordering of the atoms, this permutation invariance seems rather a desideratum than a limitation.

We note that the ability to express arbitrary antisymmetric wavefunctions is only a necessary, but not necessarily sufficient criterion for a good ansatz. Expressivity of the ansatz with a finite number of parameters, as well as stability during optimization can be of high importance in practice but are hard to assess from a purely theoretical point of view.

Supplementary references

[1] Ingrid von Glehn, James S Spencer, and David Pfau. “A Self-Attention Ansatz for Ab-initio Quantum Chemistry”. In: *The Eleventh International Conference on Learning Representations*. 2023.

[2] Jan Hermann, Zeno Schätzle, and Frank Noé. “Deep-Neural-Network Solution of the Electronic Schrödinger Equation”. In: *Nature Chemistry* 12.10 (Oct. 2020), pp. 891–897. ISSN: 1755-4349. DOI: 10.1038/s41557-020-0544-y.

[3] Michael Scherbela et al. “Solving the Electronic Schrödinger Equation for Multiple Nuclear Geometries with Weight-Sharing Deep Neural Networks”. In: *Nature Computational Science* 2.5 (May 2022), pp. 331–341. ISSN: 2662-8457. DOI: 10.1038/s43588-022-00228-x.

[4] Leon Gerard et al. “Gold-Standard Solutions to the Schrödinger Equation Using Deep Learning: How Much Physics Do We Need?” In: *Advances in Neural Information Processing Systems*. Vol. 35. Curran Associates, Inc., Oct. 2022, pp. 10282–10294.
