# Supplementary material for: Towards a transferable fermionic neural wavefunction for molecules
Source: Nat Commun. 2024 Jan 2;15:120. doi: 10.1038/s41467-023-44216-9 (PMC10762074; doi:10.1038/s41467-023-44216-9)
Supplement: Supplementary file 3 — Description of Additional Supplementary Files [file 41467_2023_44216_MOESM3_ESM.pdf]

Description of Additional Supplementary Files

Filename: fig2_HChains_extensivity_and_MottaEtAl_a
Description: Source data for figure 2a.

Filename: fig2_HChains_extensivity_and_MottaEtAl_b
Description: Source data for figure 2b.

Filename: fig2_HChains_extensivity_and_MottaEtAl_c
Description: Source data for figure 2c.

Filename: fig3_h2o_phaseflip_a
Description: Source data for figure 3a.

Filename: fig3_h2o_phaseflip_b
Description: Source data for figure 3b.

Filename: fig4_reuse_from_smaller_a
Description: Source data for figure 4a.

Filename: fig4_reuse_from_smaller_b
Description: Source data for figure 4b.

Filename: fig4_reuse_from_smaller_c
Description: Source data for figure 4c.

Filename: fig5_eval_foundational_model
Description: Source data for figure 5.

Filename: fig6_C2_PES_a
Description: Source data for figure 6a.

Filename: fig6_C2_PES_b
Description: Source data for figure 6b.

Filename: fig6_C2_PES_c
Description: Source data for figure 6v.

Filename: fig7_ablation_foundational_model_a
Description: Source data for figure 7a.

Filename: fig7_ablation_foundational_model_b
Description: Source data for figure 7b.

Filename: fig7_ablation_foundational_model_c
Description: Source data for figure 7c.
